# A Resource for Transcriptomic Analysis in the Mouse Brain

**DOI:** 10.1371/journal.pone.0003012

**Published:** 2008-08-20

**Authors:** Charles Plessy, Michela Fagiolini, Akiko Wagatsuma, Norihiro Harasawa, Takenobu Kuji, Atsuko Asaka-Oba, Yukari Kanzaki, Sayaka Fujishima, Kazunori Waki, Hiroyuki Nakahara, Takao K. Hensch, Piero Carninci

**Affiliations:** 1 Functional Genomics Technology Team, Omics Science Center, RIKEN Yokohama Institute, Yokohama, Kanagawa, Japan; 2 Laboratory for Neuronal Circuit Development, RIKEN Brain Science Institute, Wakô, Saitama, Japan; 3 Laboratory for Integrated Theoretical Neuroscience, RIKEN Brain Science Institute, Wakô, Saitama, Saitama, Japan; 4 Genome Science Laboratory, Discovery and Research Institute, RIKEN Wakô Institute, Wakô, Saitama, Japan; Temasek Life Sciences Laboratory, Singapore

## Abstract

**Background:**

The transcriptome of the cerebral cortex is remarkably homogeneous, with variations being stronger between individuals than between areas. It is thought that due to the presence of many distinct cell types, differences within one cell population will be averaged with the noise from others. Studies of sorted cells expressing the same transgene have shown that cell populations can be distinguished according to their transcriptional profile.

**Methodology:**

We have prepared a low-redundancy set of 16,209 full-length cDNA clones which represents the transcriptome of the mouse visual cortex in its coding and non-coding aspects. Using an independent tag-based approach, CAGE, we confirmed the cortical expression of 72% of the clones. Clones were amplified by PCR and spotted on glass slides, and we interrogated the microarrays with RNA from flow-sorted fluorescent cells from the cerebral cortex of parvalbumin-egfp transgenic mice.

**Conclusions:**

We provide an annotated cDNA clone collection which is particularly suitable for transcriptomic analysis in the mouse brain. Spotting it on microarrays, we compared the transcriptome of EGFP positive and negative cells in a parvalbumin-egfp transgenic background and showed that more than 30% of clones are differentially expressed. Our clone collection will be a useful resource for the study of the transcriptome of single cell types in the cerebral cortex.

## Introduction

Despite the genome sequence of the human and several other mammalian species having been determined [1]–[2], it has become evident that the transcriptional output of the genome is much more complex than the predicted protein coding genes. Although a mammalian genome contains about 20,000 protein coding genes, there are an additional 23,000 transcriptional units (TUs) comprised of RNA without protein coding potential [3], which originate from more than 181,000 different transcripts in the mouse. As detected by whole genome tiling arrays, the human genome also shows comparable complexity [4]; reviewed in [5]). EST analysis has shown that RNA expression is frequently restricted to specific tissues. For instance, more than 110,000 3′ end cDNA clusters, out of a total of 171,000 3′ end EST clusters prepared during the Mouse cDNA Encyclopedia Project, were identified from only one library [6], largely from the brain [7]. These data suggest that to comprehensively identify the RNAs involved in specific biological processes, or at least the tissue-specific RNA/mRNA isoforms, it is essential to prepare novel EST collections, preferably using normalization and subtraction to identify rarely expressed mRNAs.

Apart from the importance for expression profiling studies, full-length cDNAs are essential for the understanding of the structure of the protein encoded in specific tissues. Full-length cDNAs also constitute an invaluable resource for future functional studies such as ectopic expression with lentiviral vectors [8]. Additionally, they provide specific information for gene start and termination sites [9]. In particular, 5′ -ends help to identify tissue-specific promoter elements to connect them to TUs, and at the same time to novel datasets that allow for mRNA expression profiling based on measuring transcriptional activity at the start site (or mRNA cap). Accordingly, we have previously developed the cap-analysis gene expression (CAGE) method ([10]–[11]), which is based upon the production of short (20–21 nt) tags corresponding to the 5′ end of capped RNA transcripts. After high-throughput profiling, these are aligned onto the genome to identify their promoter elements, and the specific activity at a certain start site is measured as the frequency of CAGE tags. Full-length cDNA collections, and 5′ ESTs are very beneficial to map CAGE tags to TUs, which exhibit alternative promoter usage in at least 50% of the cases ([12]).

Here, we have developed a full-length cDNA and microarray resource for studies with particular emphasis on the mouse visual cortex. Due to tissue restriction of mRNA expression [6], we tested the resource for differential gene expression across different cell types by comparing the transcriptome of parvalbumin (*pvalb*) cells versus the rest of cortex. The *pvalb*-cell network has recently been identified as an important trigger for critical period plasticity in the postnatal visual cortex [13]. Sensory deprivation typically between postnatal day P20–P40 in mice leads to a functional and structural rewiring of visual cortical circuits that underlies an enduring loss of visual acuity (amblyopia). Direct manipulation of postnatal GABA function can delay or accelerate plasticity onset [13], carrying potentially broad implications for therapies in adulthood as well as a deeper mechanistic understanding of cognitive developmental disorders.

## Results and Discussion

We previously prepared four full-length cDNA libraries from whole extracts of wild-type mouse visual cortex at P18, P24, P28 and P55 using the CAP trapper method [14]. The libraries were subjected to subtraction and normalization [15]. A total of 76,999 5′ ends and 75,757 3′ ends were sequenced, and a cluster analysis showed that the resulting libraries had an average redundancy of 1.7 [15]. These libraries were used as a starting point for the preparation of a non-redundant clone collection representing the transcriptome of the visual cortex across its postnatal development.

**Figure 1:**
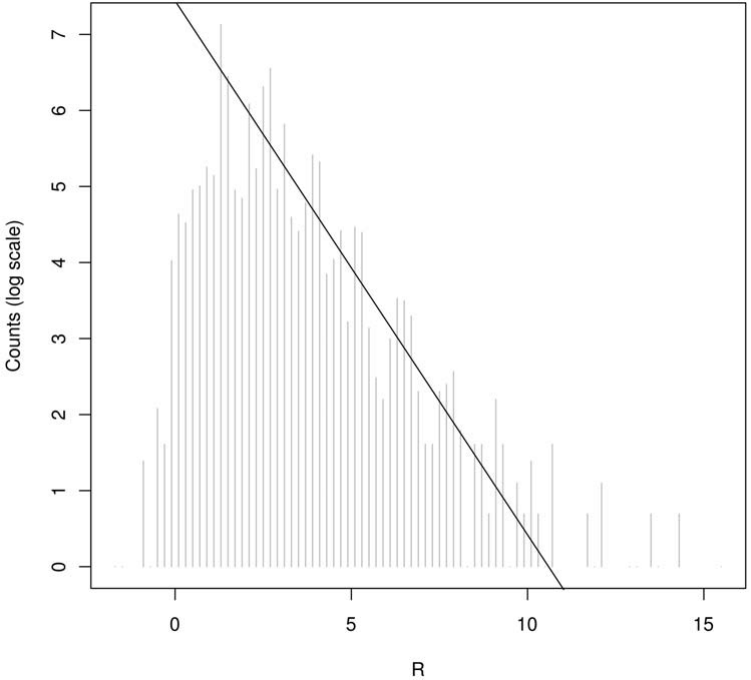
Counts in discrete intervals of R-values (natural logarithmic scale). The line was fit to the part of the curve which can be approximated by a logarithmic function. Its intersection with the x axis is suggested as a rough threshold estimate above which R-values are considered significant.

We pooled the 5′ ESTs from all four libraries and grouped them into 6,818 clusters and 9,391 singletons using the stackPACK software [16]. Thus, the redundancy increased to 4.75 after *in silico* pooling. We counted the number of 5′ ends per cluster, and identified the genes with the most imbalanced expression patterns, in order to detect potential genes of interest for the study of the maturation of visual cortex. We used the method developed by Stekel *et al.* [17] for comparing digital expression patterns, and calculated a *R* statistic for each cluster, using an in-house program available upon request. This method did not allow a quantitative prediction of false discovery rate. Hence, the upper interval of *R v*alues which cannot be fitted to an exponentially decreasing distribution were kept as potentially significant, as suggested in Stekel *et al.* [17]. We then arbitrarily selected 11 as a cut-off value (Figure 1), which highlighted fourteen protein-coding genes (Table 1).

**Table 1:** EST clusters with a strongly imbalanced developmental expression pattern.

Clone ID	*R*-value	EST count	Gene symbol	Full official name
K230007N19	15.5	43,9,23,8	Exdl2	exonuclease 3′-5′ domain-like 2
K230008D05	14.3	4,0,17,0	Kctd10	potassium channel tetramerisation domain containing 10
K330007G06	14.3	0,11,0,2	Aldh1b1	aldehyde dehydrogenase 1 family, member B1
K230004E20	13.8	26,5,16,2	Fbxw7	F-box and WD-40 domain protein 7, archipelago homolog Drosophila)
K430007K11	13.5	0,0,13,2	Kcnv1	potassium channel, subfamily V, member 1
K230005P19	13.5	22,12,1,20	Decr2	2-4-dienoyl-Coenzyme A reductase 2, peroxisomal
K330313B11	13.2	0,2,1,14	1500002O20Rik	RIKEN cDNA 1500002O20 gene
K230309E11	12.9	1,11,1,0	Ev1	Ena-vasodilator stimulated phosphoprotein
K230034K22	12.2	2,13,1,1	4632419K20Rik	RIKEN cDNA 4632419K20 gene
K230035O10	12.2	2,0,15,2	Srgap2	SLIT-ROBO Rho GTPase activating protein 3
K230025H24	12.1	2,0,16,10	Mfn2	mitofusin 2
K530003G22	12.0	0,0,0,9	Arid1b	AT rich interactive domain 1B (Swi1 like)
K230006M16	11.7	3,1,20,5	Rbm39	RNA binding motif 39
K430007G04	11.6	0,0,9,0	6330500D04Rik	RIKEN cDNA 6330500D04 gene

One representative clone was selected for each cluster. Together with the singletons, they constituted 16,209 different clones that were selected for rearray and PCR amplification in order to set up a microarray platform. 400 cDNAs were full-length sequenced and annotated as FANTOM 3 clones [3]. Of these, 179 have been annotated as non-coding by the FANTOM 3 consortium. This suggests that our whole collection is well enriched in non-coding cDNAs. To annotate the clones for which only partial sequence was available, we compared their 5′ ends to Transcription Units (TUs) of FANTOM3's Representative Transcript Set (RTS) that includes ESTs (ftp://fantom.gsc.riken.jp/RTPS/fantom3_mouse/primary_est_rtps). TUs are clusters of full-length transcripts that contain a common core of genetic information [18]. 13,189 clones whose 5′ EST was co-located to a TU inherited their annotation and identification number. The full-length cDNAs that define the TUs originate mostly from libraries made totally or partially from brain tissues (brain, whole head, whole embryo), from libraries related to the immune system (immune cells are present in most tissues and therefore contribute to their transcriptome), and from a testis library. The 3,020 clones whose 5′ end did not match a FANTOM3 TU are those whose sequence satisfied our quality criteria for clustering, but were discarded by the more stringent filters of the FANTOM3 RTS. There are 580 FANTOM 3 ESTs that are unique to our visual cortex libraries according to the FANTOM3 representative transcript set, of which 292 have been included in our spotted clone collection.

**Figure 2:**
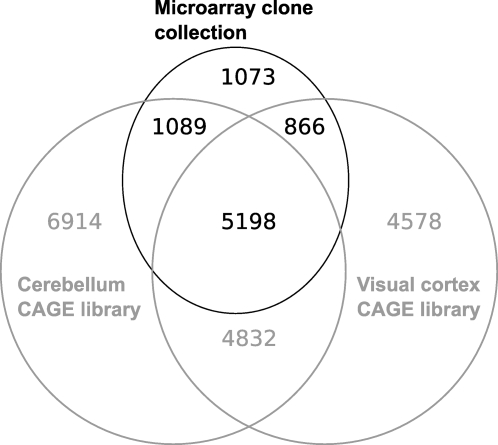
Venn diagram representing the overlap between transcription units (TUs) of our clone collection and two sets of CAGE tags originating from the cerebellum or visual cortex. Only 15% of TUs do not have a counterpart in the CAGE libraries, suggesting that our clone collection is well suited to interrogate brain samples.

To confirm the expression of our clones in the visual cortex, we prepared CAGE libraries [10]–[11] from the visual cortex of mice at four developmental stages (P21, P26, P54 and P71) and compared the overlap of the cDNA and CAGE libraries for gene detection. We mapped the CAGE tags to the mouse genome in order to assign them to TUs [12] and pooled them in one virtual library of 180,984 tags. 16,194 TUs were identified by this technique as being expressed in the visual cortex. Of these, 6,874 had a counterpart in our clone collection. Interestingly, 7,005 TUs of our collection also had a counterpart in a CAGE library of similar sequencing depth (211,541 tags) made from cerebellar tissue [12]. This suggests that our collection can also be useful in the analysis of non-cortical brain tissues (Figure 2).

Cerebral cortex is composed of many cellular and neuronal types. Our spotted clone collection represents their combined transcriptomes. We sought to investigate the complexity of a homogeneous subset of cortical cells. Parvalbumin (*pvalb*) is a calcium-binding protein which is used as a molecular signature of some GABAergic interneuron cell types [19]–[20]. Using a BAC transgenic line expressing the green fluorescent protein (EGFP) under the control of the *pvalb* regulatory sequences [21], we dissociated and sorted fluorescent and non-fluorescent cells from mouse cerebral cortex at the peak of the visual critical period (P28–29) and compared their transcriptional profiles on microarrays made with the first 4,512 clones of our collection, each spotted three times. Cy5- and Cy3-labeled cDNAs were prepared from the total RNA of 15,000–20,000 cells using the method of [22], and compared five samples using a dye-swap strategy. The data discussed in this publication have been deposited in NCBIs Gene Expression Omnibus (GEO, http://www.ncbi.nlm.nih.gov/geo/) and are accessible through GEO Series accession number GSE8968.

**Figure 3:**
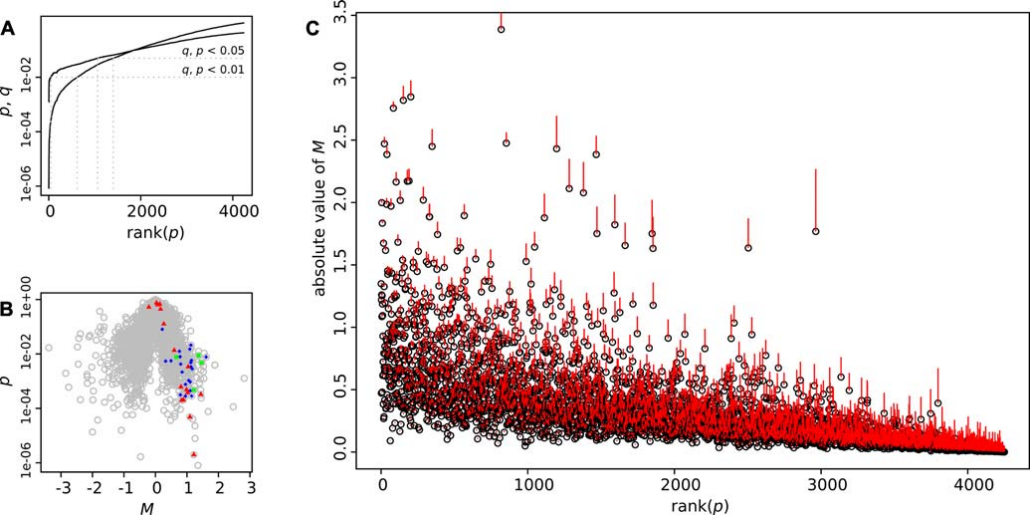
Statistical analysis of microarray results. A: q and p values ranked by p-values. B: Vulcano plot showing the p-values as a function of expression changes. Blue diamonds: genes from the Nduf family. Red triangles: genes from the Atp5 family. Green dots: genes from the Cox6 family. C: Absolute value of M ranked by increasing values of p. The red bars represent the standard deviation of M. Some genes have a low rank despite a strong M value, often because of their higher variability, as indicated by the length of their vertical bars.

The slides were scanned with the photomultiplier set to maximize signal intensity while avoiding excess saturation. The fluorescence intensity values were normalized using LOWESS normalization. To ensure data quality, we first investigated the overall quality of each slide and decided to discard the data from one slide. Second, we examined data consistency using dye-swap information within each sample and excluded clones that did not meet criterion from later statistical analysis, lowering the total number of included clones to 4,250. We ranked them by *p*-values based on a *t*-test. To address multiple comparisons, we chose *q*-values as a false discovery rate-based measure of significance [23]. By setting the significance criterion at *p*<0.05, 1,400 of the 4,250 clones were differentially expressed (*p*<0.05) (Table 2, Figure 3A). The *q*-value corresponding to criterion was 0.066, so the number of false positives was estimated to be at most 92 (92 = *q×n*).

**Table 2:** Lowest *p*- and *q*-values for given number of ranked clones, and number of clones reaching given *p*- or *q*-values.

top *n*	*p*	*q*	*n×q*	*n*	*p*	*q*	*n×q*
2000	0.12	0.11	220	1400	<0.05	0.066	92
1000	0.025	0.046	46	1062	0.029	<0.05	53
500	0.0073	0.027	13	617	<0.01	0.030	18
200	0.0019	0.017	3	46	0.00025	<0.01	0

As a control for the quality of cell sorting, we checked for genes known to be absent from *pvalb*-expressing neurons. Both GABA-synthetic enzymes, *GAD65* and *GAD67*, were included as control genes, however, they are not ideal, as *pvalb*-cells constitute only a subset of GABAergic neuron [13]. Thus, the non-*pvalb* fraction of our homogenates would include a substantial amount of *GAD* gene expression. Instead, we used *pvalb* itself as a control. Moreover, the glutamate receptor *Gria2* (*GluR2*) was significantly underrepresented in the sample prepared from fluorescent cells (*p* = 0.00048, *M* = −0.77), as expected for *pvalb*-cells that express little or no GluR2 subunits [24]. Instead, *Gria1*, another receptor from the same family that is expressed in *pvalb*-expressing cells, was over-represented in the fluorescent samples (*p* = 0.0042, *M* = 1.2).

We inspected the significantly over- or under-represented genes exhibiting the strongest expression differences between EGFP-positive and negative samples. Table 3 lists the 24 strongest variations in both directions. Prompted by the co-occurrence in Table 3 of genes implicated in ATP metabolism, such as subunits of the mitochondrial ATPase or components of the oxidative chain, we examined all other genes from these families, and found that they are particularly abundant among over-represented genes (Figure 3B, Table 4). This may reflect the intense metabolic activity of the fast-spiking *pvalb*-expressing cells [25].

**Table 3:** Clones showing the strongest statistically significant differential expression.

*under-represented*				*over-represented*			
SYMBOL	CLONE_ID	p	M	SYMBOL	CLONE_ID	p	M
Crip2	K230313M18	7.58E-3	−2.69	Cplx1	K230003P17	4.44E-3	2.58
C1qb	K230028J21	1.00E-2	−2.68	Fgf9	K230018I07	9.07E-5	2.32
Syt5	K230336L04	2.31E-3	−2.40	Gtl2	K230042M22	3.93E-3	1.88
Ctxn	K230013F20	1.93E-3	−2.33	9130213B05Rik	K230034N12	9.41E-4	1.69
Sepp1	K230009C19	4.85E-4	−2.23	6330512M04Rik	K230318F21	5.29E-3	1.62
Apoe	K230004K09	1.66E-2	−2.23	Ndufa11	K230031N16	7.69E-3	1.61
Nrn1	K230014L09	1.19E-2	−2.13	Evl	K230309E11	1.06E-2	1.55
Snca	K230001F08	1.07E-3	−2.02	Vip	K230041M20	1.25E-3	1.54
6330527O06Rik	K230003M18	2.02E-3	−2.00	Btbd14a	K330002G16	1.81E-2	1.48
6330403K07Rik	K230304J17	4.34E-4	−1.97		K330002G06	1.96E-2	1.48
D11Ertd707e	K230009N15	6.26E-5	−1.94	Cox6c	K230002F10	4.77E-3	1.46
Ptn	K230334M04	6.56E-4	−1.91	Pcdhac2	K230029N10	2.58E-3	1.45
Schip1	K230006C03	1.35E-3	−1.83	Atp5g3	K330002E13	3.25E-4	1.44
Baiap2	K230319I19	1.76E-2	−1.83	2010107H07Rik	K330010C07	1.20E-3	1.44
3110035E14Rik	K230010N04	2.01E-2	−1.82	Vamp1	K230001K20	2.35E-3	1.43
H2-DMa	K230007M04	3.35E-4	−1.78	9530058B02Rik	K230010H11	6.03E-3	1.40
Hn1	K230005H22	1.68E-4	−1.62	Cox6c	K230006O16	8.83E-3	1.37
	K230011F01	1.23E-2	−1.61	1810012P15Rik	K230027M24	1.26E-2	1.37
Ngef	K330017A06	3.67E-3	−1.51	Stac2	K230027M01	1.92E-3	1.34
Gap43	K230009L16	3.03E-2	−1.44	AI836003	K230306I16	2.78E-6	1.33
	K230302E22	2.48E-4	−1.42	Ccne1	K230044G09	1.36E-3	1.31
Fbxl2	K230024L20	5.40E-3	−1.42	Pdlim3	K230306A09	6.48E-3	1.31
Pcsk2	K230002M17	1.59E-3	−1.37	Atp1b1	K230006G24	1.56E-2	1.29
Ctsb	K230008F16	1.35E-2	−1.37	2610205H19Rik	K230306A08	7.83E-6	1.26

**Table 4:** Differential expression of the Atp5, Cox6 or Nduf family genes.

CLONE_ID	SYMBOL	p	M
K230031N16	Ndufa11	7.69E-3	1.61
K230002F10	Cox6c	4.77E-3	1.46
K330002E13	Atp5g3	3.25E-4	1.44
K230006O16	Cox6c	8.83E-3	1.37
K230309I10	Cox6b1	4.73E-4	1.22
K230001M19	Atp5h	1.99E-6	1.22
K230340G22	Ndufb9	2.84E-3	1.16
K230002E05	Ndufb8	2.84E-4	1.15
K230006I15	Ndufa8	5.80E-3	1.14
K230009H06	Ndufs5	3.36E-3	1.13
K230012O10	Ndufa2	2.13E-2	1.13
K230310N23	Ndufb7	9.34E-4	1.11
K330022I20	Ndufa6	4.48E-4	1.11
K230010M09	Atp5h	4.84E-5	1.09
K230310M07	Ndufa5	1.50E-2	1.09
K230044G12	Ndufc2	1.06E-3	1.06
K230319K23	Atp5g1	3.37E-3	1.04
K230012P07	Atp5j	3.80E-4	1.02
K230304A02	Ndufa5	8.35E-3	0.96
K230019O17	Ndufs2	2.80E-4	0.95
K230304I19	Ndufb10	7.66E-4	0.95
K330018J12	Atp5e	1.94E-3	0.92
K230039K18	Atp5c1	2.06E-4	0.89
K230050F20	Ndufb2	2.15E-3	0.85
K230053K02	Atp5o	1.98E-4	0.83
K230014B16	Atp5c1	6.17E-4	0.81
K230003F11	Ndufs8	4.23E-3	0.80
K230304N08	Ndufv2	3.12E-4	0.79
K330022A12	Ndufv1	7.91E-3	0.78
K230008K02	Ndufa10	1.25E-2	0.76
K230001C13	Cox6a1	7.63E-3	0.66
K330009A17	Atp5d	1.36E-2	0.60
K230053L04	Ndufs3	5.61E-3	0.49
K230005J19	Ndufaf1	5.43E-3	0.32
K230034B16	Atp5g2	1.27E-1	0.27
K230003B02	Ndufs1	8.05E-2	0.22
K230003P04	Atp5f1	4.42E-1	0.16
K230018C04	Atp5b	6.76E-1	0.14
K230001F17	Atp5a1	6.43E-1	0.07
K230302C16	Atp5s	7.52E-1	0.04
K230001C22	Atp5b	5.26E-1	−0.21

To confirm that the fluorescent cells consisted only of neurons, we checked for the expression of *Myelin Basic Protein* (*Mbp*). Our slides contained two different clones coding for the *Mbp* gene, both showing a strong underrepresentation (*M*≈−2.1 each). Despite this strong value, it was not prominently ranked since the *p-*value was slightly lower than 5%. These two *Mbp* clones exemplify a class of genes which display a strong variability in their differential expression across the analyzed samples (Figure 3C). Among these, we could also find another myelin-related gene, *plp1*, and a component of the MAP kinase pathway, *Map2k6*. In the case of the *Mbp* gene, we hypothesize that the source of the noise affecting the *p-*value was the quantity of oligodendrocytes included amongst the non-fluorescent cells, which can vary significantly with each dissection.

**Figure 4:**
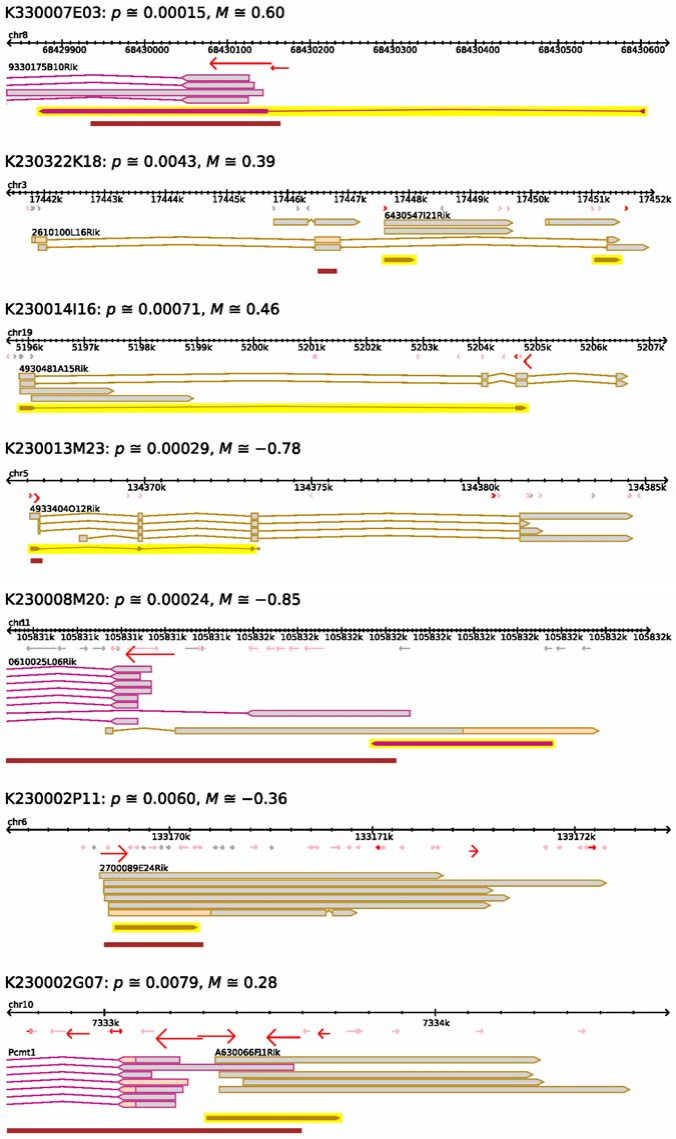
Non-coding clones which show over-representation (M>0) or under-representation (M<0) in the parvalbumin-egfp cells. Pictures extracted from the FANTOM 3 genome browser. The RIKEN clone ID is indicated, followed by the p and M values. Black rulers: chromosomal coordinates. Red arrows: known promoters. Stronger promoters are represented by bigger arrows with a stronger color. Plain arrows: known full-length cDNAs (brown boxing: plus strand; magenta boxing: reverse strand). Pink areas in the cDNAs: predicted coding sequences. The 5′ ends of the selected clones (and the 3′ end, in the case of K23022K18) are represented by plain arrows in a yellow background. K330007E03: clone overlapping the promoter of a coding gene. K230014I16: variant of a spliced non-coding gene. K230322K18: intronic clone overlapping the last exon of a coding gene. K230002G07: unspliced clone sharing the promoter of a coding gene in a head-to-head arrangement. K230002P11: intergenic clone. The coding sequence detected in one of the cDNAs is unlikely to be productive as the ATG is starting the transcript. K230013M23: spliced clone matching cDNAs with no known coding sequence. K230008M20: clone forming a sense-antisense chain with two genes oriented head-to-head.

We then sought to investigate the differential expression of non-coding transcripts. Since we do not have the full-length sequence of most of these clones, we relied on their annotation to identify those that are potentially non-coding. We reasoned that most protein-coding genes are given an original gene symbol, whereas the clones whose function was unknown keep their transient MGI (Mouse Genome Informatics) name for a long time. In our collection, these clones typically have a symbol constructed by adding “Rik” to the clone identifier. Using the FANTOM 3 genome browser, we inspected the mapping of the the top 100 clones (ranked by their *p*-value) which inherited a gene symbol including “Rik”. Only seven of these were likely to be non-coding (Figure 4).

This low occurrence of non-coding clones in the differentially regulated set can be explained as follows. First, non-coding RNAs are often weakly expressed, which makes the detection of variation in expression harder. Second, it has been reported that more than one third of all transcribed sequences exist in polyadenylated and non-poly-adenylated forms [4]. Since we used oligo dT primers during the reverse-transcription, this aggravates low expression levels. Importantly, because the function of the non-coding transcriptome is still poorly characterized, it remains unknown whether technical reasons alone or biological considerations explain the low occurrence of differentially expressed non-coding transcripts. From this perspective, our results suggest that the non-coding transcriptome is homogeneous at the level of different mature cortical populations.

We created a collection of 16,209 RIKEN clones by subtracting, normalizing and clustering cDNA libraries from the mouse visual cortex. This clone collection is representative of the brain transcriptome as confirmed by an independent transcriptional profiling using the CAGE technology. Comparison of a homogeneous interneuron population to a whole-tissue control with our cDNA microarray platform identified more than 30% of differentially expressed RNAs even taking a conservative approach. Besides full-length cDNA arrays, and the investment in equipment and resources that these technologies require, other alternatives strategies can be conceived. Possible choices are the oligonucleotide arrays. However, due to prevalence of alternative promoters, exons and polyadenylation site, one would ideally have to prepare multiple oligonucleotides for each coding and non-coding gene. Our clone collection aims at maximizing the possibility of implicating novel non-coding cDNAs in a neural function, and its advantage is that when a candidate is found, a full-length cDNA is available. For analysis focused on transcriptional control, we would rather recommend CAGE when the technology to make CAGE libraries from limited amount of tissue will be available.

The large heterogeneity of gene expression between cells strongly suggests that future studies will require the separation of single neuron populations. This is particularly true for cells that are rare in a tissue, such as the *pvalb*-expressing neurons, whose specific transcriptome variations during development and brain plasticity would be masked by the large majority of unrelated cells. In general, these data emphasize once more the importance of dissecting neuronal sub-types when expression profiling to study specific brain regions. In summary, these findings show that our clone collection, assembled with an unbiased and hypothesis-free strategy, is suitable for probing the transcriptome of purified cell populations and is broad enough to provide control genes. Our publicly available cDNAs are thus a proven tool for the profiling and comparison of homogeneous cell types within the brain. This will help progress toward better defining these cell types at a transcriptomic level [26]–[27].

## Materials and Methods

### Sequencing and clustering

76,999 5′ ESTs from the K2, K3, K4 and K5 libraries were sequenced with RISA sequencers [28] and deposited in DDBJ under the accession numbers BY237236–BY298532 (all the range), CJ142844–CJ159349 (a subset of the range). 367 additional ESTs were used but not deposited in DDBJ because they were superseded by higher quality reads for the same clone. Their sequence is available upon request. The 5′ ESTs were clustered using stackPACK [16] with the following external programs: d2_cluster [29] (word_size = 6, similarity_cutoff = 0.98, minimum_sequence_size = 50, window_size = 200, reverse_comparison = 1), phrap (P. Green, unpublished) (old_ace = 1, vector_bound = 0, forcelevel = 5, trim_score = 20, penalty = −2, gap_init = −4, gap_ext = −3, ins_gap_ext = −3, del_gap_ext = −3, maxgap = 30, flags = -retain_duplicates -node_space 8) and craw [30] (sig = 0.5, window_size = 100, ignore_first = 50). In addition, 75,757 3′ ESTs were deposited in DDBJ under the accession numbers BY599749–BY660055 and CJ279459–CJ294908.

### Annotation

The annotation of full-length cDNAs which were sequenced after completion of the FANTOM3 project was made in a pipeline containing five filters (Figures S1 and S2). First, the FANTOM3 representative transcript set [3] and refSeq [31] were searched for identical DNA sequences (> = 98% similarity over > = 100 bp) using the fasta34 program from the fasta3 package [32] with the parameters -Q -H -n -d0 -m9 -E100. Clones with no identical match were searched for open reading frames identical or similar to the entries of Swiss-Prot [33], TrEMBL [33] or refSeq using the fasty34 program from the fasta3 package, with the parameters -Q -H -n -d0 -m9 -E100. cDNA clones with no match were scanned for potential open reading frames (ESTs were skipped) using the DECODER [34], geneid 1.2 [35] and orfind (Tatusov, unpublished) programs. ESTs were compared to the UniGene database [36] using fasta34 with the same parameters. Clones which were not caught by any of these filters were mapped on the mouse genome (release UCSC mm5 [2]) using the sim4 [37] program.

### CAGE analysis

Cage libraries were prepared as in [10], using the visual cortex of mice at age P21, P26, P54 and P71. The libraries were sequenced and analyzed as in [12], and were deposited in DDBJ under the accession numbers AAAAN0000001–AAAAN0074253, AAAAI0000001–AAAAI0019356, AAAAM0000001–AAAAM0054286 and AAAAK0000001–AAAAK0041805 respectively. The cerebellar CAGE library is available under the accession numbers AAAAA0000001–AAAA0240780.

### Rearray and clone amplification

cDNA clones were rearrayed with a Q-bot instrument (Genetix) from 20% glycerol/LB ampicillin stocks that were kept at −80°C. A growth check was performed on all glycerol stocks. For the growth check, 384-well plates were translated into four 96-well plates per 384-well plate and one bacterial culture per glycerol stock was inoculated. All plates were analyzed after 18 h of growth at 37°C by visual inspection. We performed one Plasmid DNA Preparation per Glycerol Stock using a Montage Purification Kit (Millipore) and our robotic systems. One quality control was performed on three clones per plate, and the yield was 2.66 µg DNA±1.73. For the preparation of PCR templates, we diluted the plasmid DNA preparation by 1∶500. 5 µl of template were amplified in 50 µl reactions in 96-well plates with 1.25 U Expand High Fidelity Taq/Tgo polymerase blend (Roche), 15 pmol of forward primer ( tgtaaaacgacggccagt ), 15 pmol of reverse primer ( agcggataacaatttcacacagga ) and 200 µM of each dNTP (TaKaRa) in 1× Expand High Fidelity buffer (Roche). The program of the thermocycler was 2 min at 94°C followed by 30 cycles of 15 s at 94°C, 30 s at 60°C, and 2 min 30 s at 68°C, plus a final step of 7 min at 72°C. The amplification products were then purified with Multiscreen PCR plates (Millipore) following manufacturer instruction, and dried. With this protocol, we could achieve an average yield of >8 µg for 92.7% of the wells.

### Animals and tissue preparation

We used C57Bl/6 BAC transgenic mice expressing the enhanced green fluorescent protein (EGFP) under the control of the *parvalbumin* regulatory sequences [21]. Animals were maintained on a 12 h light/dark cycle with access to food and water *ad libitum*. All procedures involving animals and their care were carried out in accordance with the directives of RIKEN's Institutional Animal Care and Use Committee. All experimental groups were sacrificed at a similar time of day to avoid possible circadian effects. Primary visual cortex (area V1) from anesthetized mice was dissected and homogenized by sonication.

### Microarray analysis

The PCR-amplified cDNAs were resuspended in 50% DMSO (Nacalai Tesque, Kyoto, Japan), and microarrays were printed on UltraGAPS slides (Corning) using an OmniGrid arrayer (Genomic Solutions) with Microarray Stealth Spotting pins (SMP3B, TeleChem). Detailed information about clone names, coordinates and accession numbers have been deposited in NCBIs Gene Expression Omnibus (GEO, http://www.ncbi.nlm.nih.gov/geo/) and are accessible through GEO Platform accession number GPL5681.

Total RNA was extracted from 15,000–20,000 FACS-sorted cells (Wagatsuma *et al,* in prep.) using the RNEasy Mini kit from Qiagen according to the manufacturer's instructions, and stored at −80°C until use.

The following protocol is for one slide, and was scaled accordingly if more than one slide was to be hybridized. Total RNA samples were mixed with 2 µl of SPIKE Mix (GE Healthcare Lucidea universal scorecard), and probes were prepared following the method of [22], except that 3 µg of cDNA were used per slide, and that the probes were dried and resuspended in 6 µl of water. The probes were then denatured at 95°C for 2 min and then mixed with 7.5 µl 4× Microarray hybridization solution version 2 (Amersham, Cat. No RPK0325), 15 µl formamide, and 145 µl of hybridization buffer (2.5 ng/µl oligo(dT) 12–18 primers (Invitrogen), 500 ng/µl mouse Cot-1 DNA (Invitrogen), 0.5× Microarray hybridization buffer version 2 (Amersham, Cat. No RPK0325, stock concentration: 4×), 25% formamide (Nacalai Tesque), 50% ULTRAhyb buffer (Ambion)), at 50°C.

The mixture of Cy3- and Cy5-labeled probes was applied at 75°C onto arrays, and hybridized for 3 h at 65°C, 3 h at 55°C and 12 h at 50°C in the dark, using an automatic hybridizer, GeneMachines HybStation (Genomic Solutions). After hybridization, slides were washed once in 2× saline-sodium citrate (SSC) and 0.2% SDS at 53°C, in 1× SSC and 0.2% SDS at 53°C, in 2× SSC at 24°C, and in 0.2× SSC for 5 min at 24°C using one cycle of 10 s flow and 1 s hold, and 15 (first wash) or 13 (other washes) cycles of 1 s flow and 10 s hold, and then dried by spinning for 1 min.

The scanning was done using an Axon GenPix 4000B scanner (Axon Instruments, Union City, CA) at 10 µm resolution. PMT voltage settings were varied to obtain maximum signal intensities with <5% probe saturation. TIFF images were captured and analyzed with GenePix 6.0 (Axon Ins.) software and Acuity 4.0 (Axon Ins.). We used the Autoflag function to exclude spots for which the signal intensity was not higher than the local background. In addition, we also flagged the spots for which the scanning area was lower than 100 pixels. We call “standard” the experiments in which the probes prepared from the fluorescent cells were labelled with Cy5, and “swapped” the ones where the label was Cy3.

### Statistical analysis

Lowess (locally weighted scatter plot for smoother) analysis was used for normalizing microarray data. Within-print-tip-group normalization was conducted by using the loess.smooth function in the statistical software package R [38]. In short, this lowess normalization approach is to use the dependence of *M = log_2_ (Cy5/Cy3)* on *A = log_2_ (Cy5×Cy3)* for estimating its normalizing curve, which is a function of *A* [39]–[40]. Due to its estimation procedure, its normalization curve is considered as estimated mostly based on unexpressed clones (which should be the majority) in each small segment of *A* [40] (we used 50 segments). We tried to further ensure this point in our lowess normalization; for each segment of *A* we defined a window that included 40% of the points that are nearest. Inside this window, the standard deviation of *M* values was calculated and lowess normalization curve was estimated by using only data points whose *M* values fell into a range within three times the standard deviation in each *A* segment. Similar results could be obtained using smaller cutoff values, e.g. 1 s.d., suggesting that this three s.d. is stringent enough (data not shown). Once the normalization curve was estimated, data points out of the range were also normalized.

To ensure data quality, we then excluded some clones from later statistical analysis by using the following two criteria, called global filtering and individual filtering. For global filtering, overall quality of each sample was examined using 3 repeated spottings (called replicates and denoted by rep1, rep2, rep3 below). For each standard or swap dataset (of each sample), the correlation of *M* values was calculated between replicates (e.g. the correlation between rep1 and rep2) and averaged over three cases (i.e. rep1 vs. rep2, rep2 vs. rep3, and rep3 vs. rep1). In all standard and swap datasets except the sample 2 standard dataset, the averaged correlation was larger than 0.899, whereas it was only 0.663 in the sample 2 standard dataset. We inspected the sample 2 standard dataset by creating the color image of *M* values in the format of the original microarray plate, and found blurred traces largely stretched over the plate. Given these, we decided to discard the sample 2 standard dataset from later statistical analysis.

Next, for individual filtering, we examined the clone-wise consistency between standard and swap datasets (within each sample). We used the lowess-adjusted *M* values for this purpose and first obtained the mean of the adjusted *M* values over replicates for standard and swap datasets, denoted by 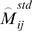 and 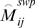, respectively, where *i* and *j* are the indices of clones and samples, respectively, *i.e. i = 1,…4541*, *j = 1,…5*. Second, for each sample (each fixed *j*), ideally we expect a consistency such that 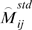 is nearly equal to 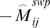, where the multiplier −1 is necessary because of the swapped dye labeling. Hence, for each sample (each fixed *j*), we calculated the standard deviation of 
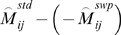
 over clones (*i.e.* over the index *i*). Clones were flagged, if they were outside the range of two times the standard deviation; these flagged clones were excluded from later statistical analysis.

We used two statistics, *p*-value for identifying differentially expressed genes and *q*-value for dealing with issues of multiple hypotheses testing, *i.e.* controlling false discovery rate (see below). In calculating these, dye-averaged value was used for each clone, given by multiplying −1 to 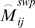 as 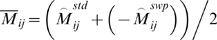. Note that due to the filtering analysis described above, sample 2-standard dataset and also clones flagged by individual filtering were excluded from the calculation. In cases when the number of samples for each clone became less than 3 due to the above filtering or some other bad conditions, the clone was excluded from calculation to secure further robustness. *p*-value was obtained by calculating *t*-statistics 
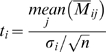
 with *n−1* degrees of freedom, where *n* was the number of samples taken into account for calculating the mean of each clone. *q*-value can be used to assess false discovery rate (FDR; more precisely, pFDR, see [41] for details). For this calculation, we used QVALUE software at http://genomine.org/qvalue/. In short, for a specified threshold *r* (*0<r≤1*), we can write 
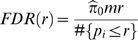
, where *m* indicates the number of *p_i_*, and 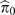 is estimated by 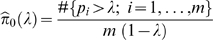 where *λ* is a tuning parameter to adjust the degree of conservative estimate of FDR and *q* value [23]. We used cubic spline at *λ = 1* and the *q* value is estimated by 
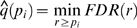
.

## Supporting Information

Figure S1 Annotation pipeline (databases).
(0.06 MB PNG)

Figure S2 Annotation pipeline (scores).
(0.09 MB PNG)
