# Supplementary figures and images for: A Resource for Transcriptomic Analysis in the Mouse Brain
Source: PLoS One. 2008 Aug 20;3(8):e3012. doi: 10.1371/journal.pone.0003012 (PMC2507754; doi:10.1371/journal.pone.0003012)

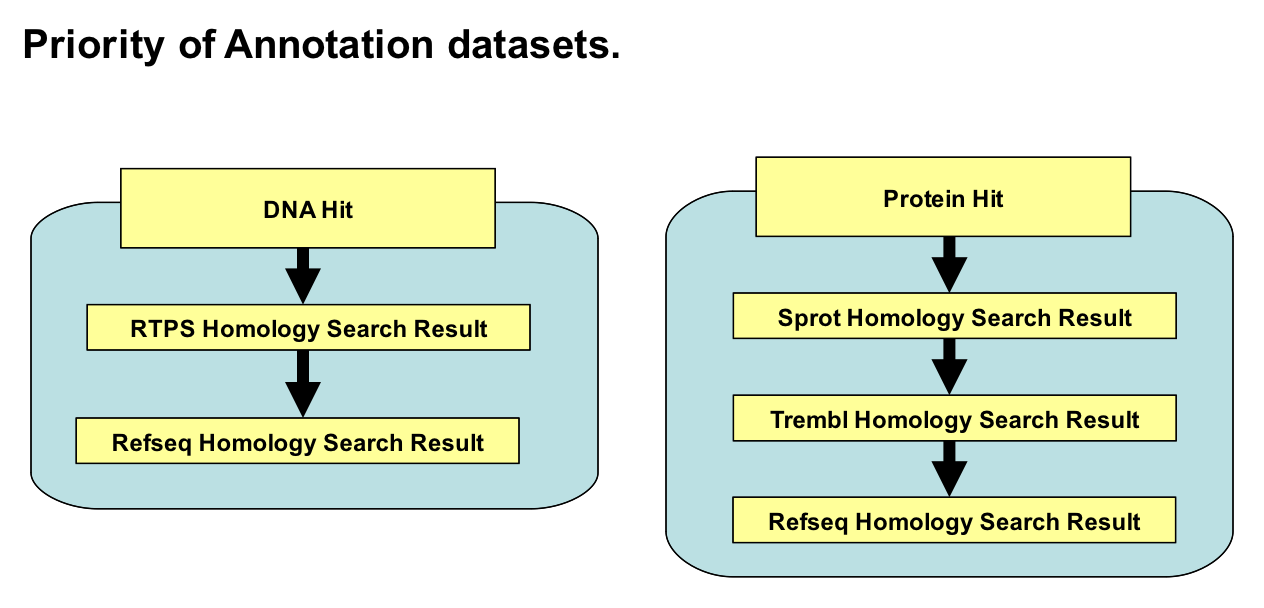

Supplement: Figure S1 — Annotation pipeline (databases). (0.06 MB PNG) [file pone.0003012.s001.png]

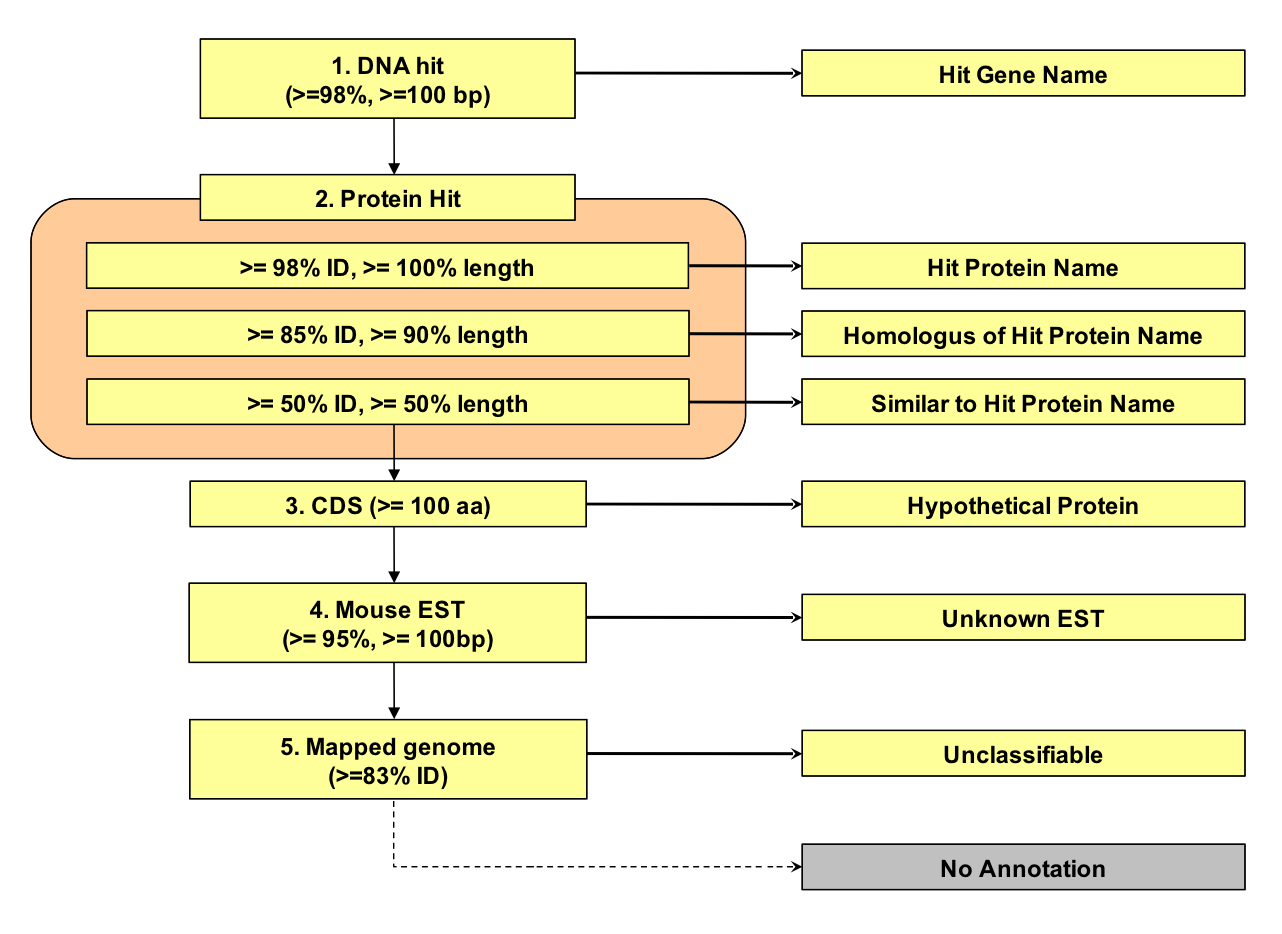

Supplement: Figure S2 — Annotation pipeline (scores). (0.09 MB PNG) [file pone.0003012.s002.png]
